# Impact of ultrasound-guided nerve block on postoperative delirium in elderly patients undergoing diabetic foot surgery: a propensity score-matched retrospective study

**DOI:** 10.3389/fnagi.2026.1801575

**Published:** 2026-08-12

**Authors:** Zhu Wen, JiaLi Zheng, Jing Huang, Juan Li

**Affiliations:** 1 Mianyang Key Laboratory of Anesthesia and Neuroregulation, Mianyang, China; 2 Department of Anesthesiology, Mianyang Central Hospital, Mianyang, China; 3 Department of Ultrasound Medicine, Mianyang Central Hospital, Mianyang, China; 4 Department of Endocrinology and Metabolism, Mianyang Central Hospital, Mianyang, China

**Keywords:** diabetic foot, neuroinflammation, opioid-sparing effect, postoperative delirium, propensity score matching, ultrasound-guided nerve block

## Abstract

**Background:**

Postoperative delirium (POD) is a common and serious complication in elderly patients undergoing diabetic foot surgery, often linked to excessive opioid use and systemic neuroinflammation. This study aimed to evaluate whether ultrasound-guided nerve block (NB) reduces the incidence of POD compared to general anesthesia (GA) in this high-risk population.

**Methods:**

We conducted a propensity score-matched (PSM) retrospective cohort study involving 137 patients. The primary outcome was the incidence of POD, while secondary outcomes included intraoperative opioid consumption (sufentanil equivalent) and postoperative interleukin-6 (IL-6) levels. Standardized mean differences (SMDs) were used to assess covariate balance after matching.

**Results:**

After PSM, the incidence of POD was significantly lower in the NB group (5.6%) than in the GA group (25.0%, *P* = 0.046). NB significantly reduced intraoperative sufentanil requirements (*P* < 0.001) and suppressed the elevation of IL-6 at 24 h postoperatively (*P* < 0.001). The protective association with NB was directionally consistent across crude, multivariable-adjusted, and matched analyses. No significant differences were observed in postoperative CRP levels or length of hospital stay (*P* > 0.05).

**Conclusion:**

Ultrasound-guided NB is associated with a lower POD risk in elderly patients with diabetic foot, consistent with potential mediation through opioid-sparing and anti-inflammatory mechanisms.

## Introduction

Postoperative delirium (POD) is a frequent and clinically consequential neurocognitive complication after surgery, characterized by an acute and fluctuating disturbance of attention and cognition that is associated with prolonged hospitalization, functional decline, and increased mortality (Marcantonio, 2017). Contemporary perioperative guidelines emphasize that POD is often preventable through early identification of high-risk patients and mitigation of precipitating factors, including excessive surgical stress, uncontrolled pain, sleep disruption, and iatrogenic exposure to deliriogenic medications (Aldecoa et al., 2017, 2024). Despite growing awareness, POD remains under-recognized in high-risk surgical populations where multimorbidity and physiological vulnerability converge—settings in which optimizing anesthetic and analgesic strategies may offer a pragmatic leverage point to reduce delirium burden (Aldecoa et al., 2017, 2024).

Patients undergoing surgery for diabetic foot disease represent a particularly susceptible group. Diabetes has been associated with a higher overall risk of delirium, and emerging evidence suggests that preoperative diabetes may independently increase POD risk even after extensive adjustment for confounders (Komici et al., 2025; Shang et al., 2024; Wei et al., 2026). In diabetic foot surgery, the typical coexistence of peripheral vascular disease, neuropathy, infection, renal dysfunction, and frailty creates a “perfect storm” for perioperative neuroinflammation and hemodynamic instability—mechanisms repeatedly implicated in POD pathogenesis (Aldecoa et al., 2024; Wang et al., 2022). Meanwhile, pain and opioid exposure are modifiable triggers: inadequate analgesia and opioid-related adverse effects may amplify sleep disturbance and neurocognitive vulnerability, reinforcing the rationale for analgesic approaches that provide stable intraoperative conditions while minimizing systemic sedatives and opioids (Marcantonio, 2017; Aldecoa et al., 2024).

Regional techniques, particularly ultrasound-guided peripheral nerve blocks (PNB), may address several delirium-relevant pathways simultaneously—attenuating the stress response, improving analgesia, reducing opioid requirements, and facilitating hemodynamic stability (Aldecoa et al., 2017, 2024). Yet the delirium benefit of regional vs. general anesthesia has been inconsistent across broader surgical populations, with large randomized evidence in hip-fracture surgery showing no significant reduction in POD under certain anesthetic and sedation strategies (Li et al., 2022; Neuman et al., 2021). Meta-analyses likewise report heterogeneity, suggesting that the effect may be context-dependent and influenced by patient selection and perioperative co-interventions (Zhu et al., 2022). Against this backdrop, diabetic foot surgery may be a setting where the physiologic advantages of PNB are more pronounced: recent clinical studies comparing PNB with general anesthesia in diabetic foot operations have reported favorable intraoperative stability and postoperative recovery profiles (Zhu et al., 2023; Zou et al., 2024). Furthermore, given the central role of inflammation in POD and the potential delirium-modulating effects of perioperative sedative/analgesic choices, integrating a robust adjustment strategy (e.g., propensity score methods) could help clarify whether ultrasound-guided nerve block is associated with a lower delirium risk in real-world diabetic foot surgery (Wang et al., 2022; Flukiger et al., 2018).

## Methods

### Study design and ethical approval

This retrospective cohort study was conducted at a tertiary teaching hospital in accordance with the principles of the Declaration of Helsinki. The study protocol was reviewed and approved by the Biomedical ethics committee of Mianyang Central Hospital (Approval No. S202503208-01). The ethical approval covered the retrospective analysis of anonymized clinical data collected between October 2022 and October 2025. Given the retrospective nature of the study and the use of de-identified data, the requirement for written informed consent was waived by the ethics committee.

Adult patients with a diagnosis of diabetic foot disease who underwent surgical treatment between October 2022 and October 2025 were consecutively screened for eligibility. Diabetic foot disease was defined according to established clinical criteria, including the presence of diabetes mellitus with concomitant foot ulceration, infection, or ischemic lesions requiring operative intervention. Eligible surgical procedures included debridement, drainage, limited amputation, or other limb-salvage operations performed below the knee level.

### Inclusion and exclusion criteria

Patients were included if they were aged 60 years or older, had a confirmed diagnosis of diabetes mellitus, and underwent surgery under either ultrasound-guided peripheral nerve block anesthesia or general anesthesia. The lower age threshold of 60 years was applied because diabetic foot disease requiring surgical intervention predominantly affects older adults, and this population carries the highest known vulnerability to postoperative delirium. Patients were excluded if they had documented preoperative delirium or severe cognitive impairment (defined operationally as a clinical diagnosis of dementia or a prior episode of delirium documented in the preoperative anesthesia record within the institutional electronic medical record system), a history of major psychiatric illness, central nervous system disease affecting cognition, emergency surgery, combined anesthesia techniques, or incomplete perioperative clinical records. When patients underwent more than one operation during the study period, only the first eligible procedure was included to avoid within-patient correlation.

### Anesthesia management

The choice of anesthesia technique was determined by the attending anesthesiologist based on clinical judgment, patient comorbidities, and surgical requirements. In the nerve block group, ultrasound-guided peripheral nerve block anesthesia was performed using a high-frequency linear transducer. Depending on the surgical site, femoral nerve block, sciatic nerve block, or a combined femoral–sciatic nerve block was administered. Local anesthetics were injected under real-time ultrasound guidance to ensure accurate perineural spread. Light sedation was permitted at the discretion of the anesthesiologist, typically using low-dose midazolam or propofol infusion, while maintaining spontaneous ventilation.

In the general anesthesia group, anesthesia was induced with intravenous agents and maintained with inhalational or total intravenous anesthesia according to institutional protocols. Endotracheal intubation or supraglottic airway devices were used as clinically indicated. Intraoperative analgesia commonly included opioids, and vasoactive agents were administered when necessary to maintain hemodynamic stability. Standard monitoring was applied in both groups, including continuous electrocardiography, non-invasive blood pressure measurement, pulse oximetry, and end-tidal carbon dioxide monitoring when applicable.

### Perioperative data collection

Demographic data, including age and sex, were extracted from electronic medical records. Preoperative clinical variables included body mass index, duration of diabetes, glycated hemoglobin (HbA1c), comorbidities (such as hypertension, coronary artery disease, and chronic kidney disease), and American Society of Anesthesiologists (ASA) physical status classification. Laboratory indices reflecting inflammatory status, including C-reactive protein (CRP) and interleukin-6 (IL-6), were collected at 24 h postoperatively as part of routine perioperative laboratory monitoring. Preoperative CRP values were extracted from the most recent available blood test obtained within 48 h prior to surgery.

Intraoperative variables included duration of surgery, estimated blood loss, use and dosage of opioids (converted to sufentanil equivalents), use of vasoactive drugs, and intraoperative hemodynamic events. Postoperative variables included pain scores assessed by standard numerical rating scales, length of hospital stay, and postoperative complications.

### Assessment of postoperative delirium

Postoperative delirium was the primary outcome of interest. Delirium assessment was performed by trained ward nurses who had completed standardized instruction in the Confusion Assessment Method (CAM) prior to the study period. Assessments were conducted once daily during a fixed morning window (08:00–10:00) on postoperative days 1 through 3. In addition, if nursing staff observed acute behavioral changes or fluctuating cognition outside the scheduled window, a supplementary CAM assessment was performed and documented. Given the retrospective design, assessors were not blinded to the patients' anesthesia technique, which represents a potential source of ascertainment bias that is acknowledged in the Discussion. Delirium was defined as an acute onset of fluctuating disturbances in attention and awareness accompanied by cognitive changes not better explained by a preexisting neurocognitive disorder. Cases of transient postoperative agitation without meeting formal diagnostic criteria were not classified as delirium.

### Propensity score matching

Given the non-randomized nature of anesthesia selection, propensity score matching was used to minimize baseline confounding between the nerve block and general anesthesia groups. Propensity scores were estimated using a multivariable logistic regression model incorporating clinically relevant pre-treatment covariates, including age, sex, body mass index, HbA1c, ASA classification, comorbidities (hypertension, coronary artery disease, chronic kidney disease), preoperative CRP, and operative duration. Intraoperative variables that are consequences of the anesthetic technique (e.g., sufentanil consumption) were not included in the propensity model. Patients were matched 1:1 using a nearest-neighbor approach with a caliper width of 0.2 standard deviations of the logit of the propensity score, without replacement.

**Figure 1:**
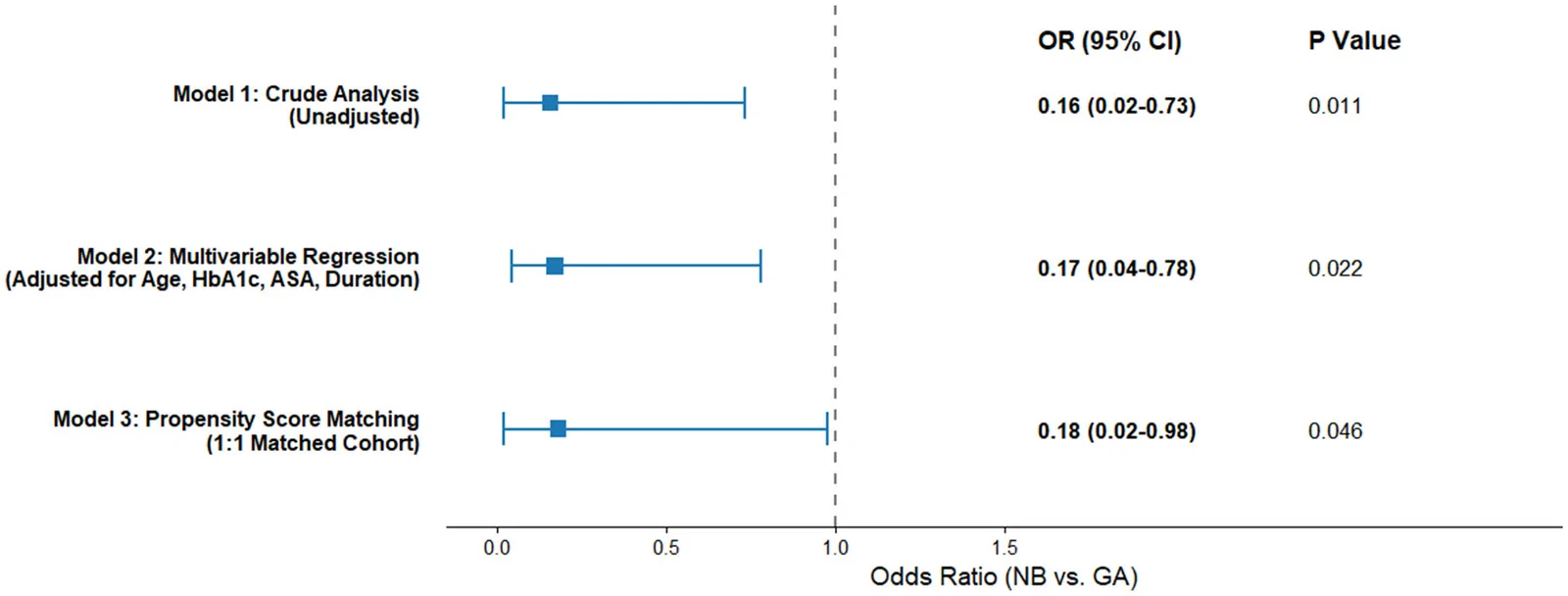
Association between anesthesia technique and postoperative delirium across different analytical models.

Covariate balance before and after matching was assessed using standardized mean differences (SMDs), with values below 0.10 indicating adequate balance; these values are reported for every covariate in Table 1. After matching, categorical covariates (sex, ASA status, hypertension, coronary artery disease, and chronic kidney disease) were well-balanced (SMD ≤ 0.05), whereas a residual imbalance persisted in preoperative CRP (SMD = 0.83) and, to a smaller degree, in age, body mass index, and operative duration (SMD 0.18–0.29). To address potential residual confounding, the association between anesthesia technique and delirium was examined across multiple analytical models (crude, multivariable-adjusted, and matched; see Statistical analysis and Figure 1), and the residual imbalance is explicitly acknowledged in the Limitations.

**Table 1:** Baseline demographic and clinical characteristics after propensity score matching.

Characteristic	GA group (*n* = 36)	NB group (*n* = 36)	*P* value	Test	SMD
Age, years (mean ± SD)	70.61 ± 6.67	68.89 ± 5.78	0.245	*t*-test	0.28
Sex, *n* (%)			1	χ^2^	0.00
Male	23 (63.9)	23 (63.9)			
Female	13 (36.1)	13 (36.1)			
BMI, kg/m^2^ (mean ± SD)	24.93 ± 3.02	25.49 ± 3.34	0.46	*t*-test	0.18
HbA1c, % (mean ± SD)	8.18 ± 1.04	8.36 ± 1.34	0.524	*t*-test	0.15
ASA physical status, *n* (%)			0.964	χ^2^	0.05
ASA II	9 (25.0)	10 (27.8)			
ASA III	25 (69.4)	24 (66.7)			
ASA IV	2 (5.6)	2 (5.6)			
Hypertension, *n* (%)—yes	24 (66.7)	24 (66.7)	1	χ^2^	0.00
Coronary artery disease, *n* (%)—yes	6 (16.7)	6 (16.7)	1	χ^2^	0.00
Chronic kidney disease, *n* (%)—yes	5 (13.9)	5 (13.9)	1	χ^2^	0.00
Preoperative CRP, mg/L (median [IQR])	18.66 [14.27, 21.84]	14.12 [11.83, 18.97]	0.029	Mann–Whitney U	0.83
Operative duration, min (mean ± SD)	95.09 ± 24.04	89.02 ± 17.31	0.223	*t*-test	0.29
Sufentanil consumption, μg (median [IQR])	27.20 [21.73, 28.91]	8.56 [6.85, 10.01]	< 0.001	Mann–Whitney U	4.54

Values are presented as mean ± standard deviation (SD), median [interquartile range], or number (percentage), as appropriate. P values were calculated using Student's t test, chi-square test, or Mann–Whitney U test, as appropriate. GA, general anesthesia; NB, nerve block anesthesia; BMI, body mass index; ASA, American Society of Anesthesiologists; CRP, C-reactive protein; SMD, standardized mean difference (calculated as the absolute difference in group means or proportions divided by the pooled standard deviation). An SMD below 0.10 indicates adequate post-matching covariate balance; covariates exceeding this threshold (preoperative CRP, age, body mass index, operative duration) showed residual imbalance that is acknowledged in the Limitations and examined through the multi-model analyses in Figure 1. Sufentanil consumption is a post-treatment variable shown for descriptive purposes and was not included in the propensity score model or treated as a balance target.

### Statistical analysis

Continuous variables were summarized as mean ± standard deviation or median with interquartile range, depending on distribution normality, while categorical variables were expressed as counts and percentages. Group comparisons were performed using Student's *t*-test or the Mann–Whitney *U* test for continuous variables and the chi-square test or Fisher's exact test for categorical variables, as appropriate. Multivariable logistic regression analysis was conducted in the matched cohort to evaluate the association between anesthesia technique and postoperative delirium, with results presented as odds ratios and 95% confidence intervals.

To assess the robustness of the association between anesthesia technique and postoperative delirium against residual confounding, the estimate was compared across complementary analytical strategies: an unadjusted analysis and a multivariable-adjusted analysis in the full unmatched cohort, and the propensity score-matched analysis (Figure 1). Concordance of effect direction and magnitude across these models was interpreted as supporting robustness. All statistical analyses were conducted using R software, and a two-sided *p* value of less than 0.05 was considered statistically significant.

### Data integrity and reproducibility

All data extraction and analysis procedures were independently verified by two investigators to ensure accuracy and consistency. Any discrepancies were resolved through consensus review of the original medical records. The analytical code and statistical workflow were archived to ensure reproducibility of the results.

## Results

### Study population and cohort construction

**Figure 2:**
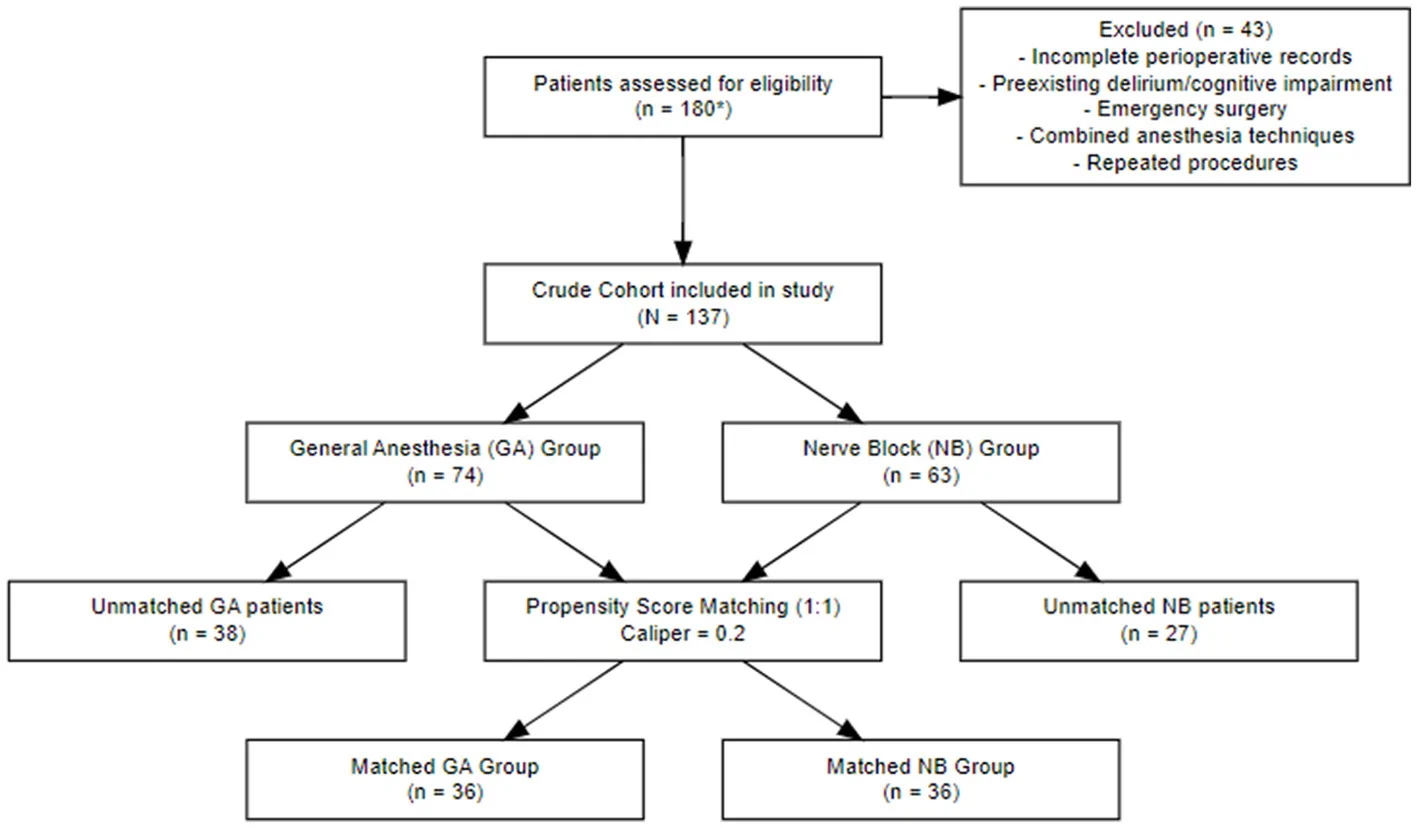
Flow diagram of patient selection and propensity score matching.

A total of 180 patients were initially assessed for eligibility during the study period. After excluding 43 patients who did not meet the inclusion criteria—primarily due to incomplete perioperative records, preexisting delirium or severe cognitive impairment, emergency surgery, combined anesthesia techniques, or repeated surgical procedures −137 adult patients undergoing surgery for diabetic foot disease were included in the crude cohort. Among these, 74 patients received general anesthesia (GA) and 63 received ultrasound-guided peripheral nerve block (NB). Following 1:1 propensity score matching using a nearest-neighbor approach with a caliper of 0.2 standard deviations of the logit of the propensity score (without replacement), a final analytical cohort of 72 patients (36 pairs) was generated for the primary outcome analysis (Figure 2). All matched patients had complete data for the primary outcome, ensuring that no post-matching attrition occurred.

### Baseline characteristics and perioperative variables

Baseline demographics and perioperative characteristics of the propensity score-matched (PSM) cohort, together with standardized mean differences (SMDs), are summarized in Table 1. After matching, the GA and NB groups were balanced with respect to sex, ASA physical status, and major comorbidities (all SMD ≤ 0.05), and were comparable in HbA1c. Residual differences nonetheless persisted for several continuous covariates, most notably preoperative CRP (SMD = 0.83), and to a lesser extent age, body mass index, and operative duration (SMD 0.18–0.29). Because these exceeded the pre-specified balance threshold of 0.10, their potential influence on the treatment-effect estimate is examined through the multi-model analyses described below (Figure 1) and explicitly acknowledged in the Limitations.

**Figure 3:**
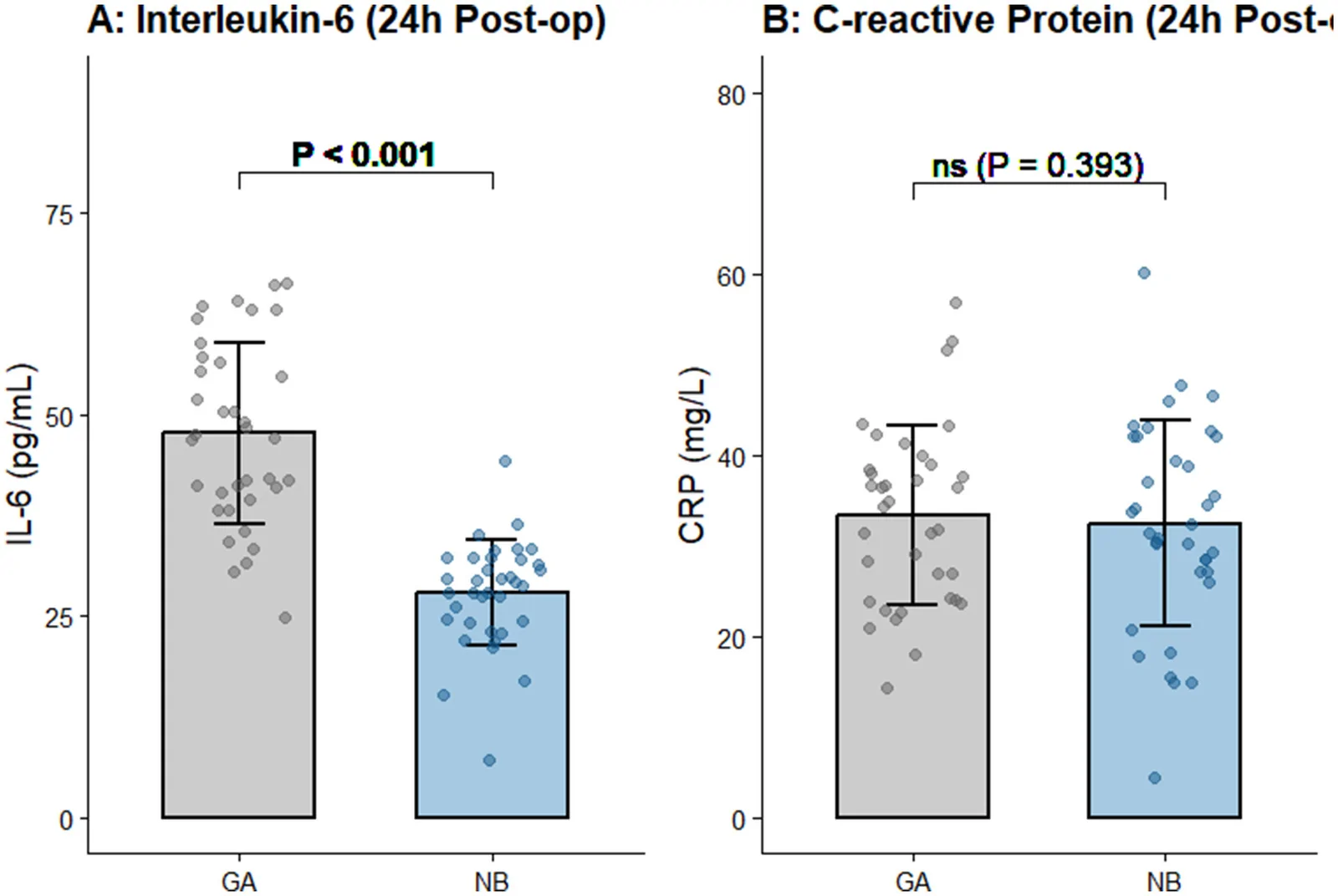
Comparison of perioperative inflammatory markers at 24 h postoperatively between anesthesia groups. **(A)** Interleukin-6 (IL-6) levels. **(B)** C-reactive protein (CRP) levels.

Distinct differences in anesthetic management and inflammatory responses were observed between the two groups. Intraoperative opioid consumption (sufentanil-equivalent dose) was significantly lower in the NB group than in the GA group (median [IQR]: 8.56 [6.85, 10.01] vs. 27.20 [21.73, 28.91] μg, *P* < 0.001). This reduction was achieved without prolonging operative time (89.02 ± 17.31 vs. 95.09 ± 24.04 min, *P* = 0.223) or increasing the requirement for vasoactive support. Postoperative inflammatory markers exhibited a divergent pattern: interleukin-6 (IL-6) levels at 24 h postoperatively were significantly lower in the NB group (27.14 ± 7.92 vs. 46.82 ± 11.35 pg/mL, *P* < 0.001), whereas C-reactive protein (CRP) levels showed no significant intergroup difference (*P* = 0.393; Figure 3). These findings suggest that while NB anesthesia effectively modulates the early cytokine cascade—represented by IL-6—it may not lead to a uniform attenuation of all systemic inflammatory pathways, such as the downstream hepatic acute-phase response (CRP), within the assessed time window.

### Incidence of postoperative delirium

**Figure 4:**
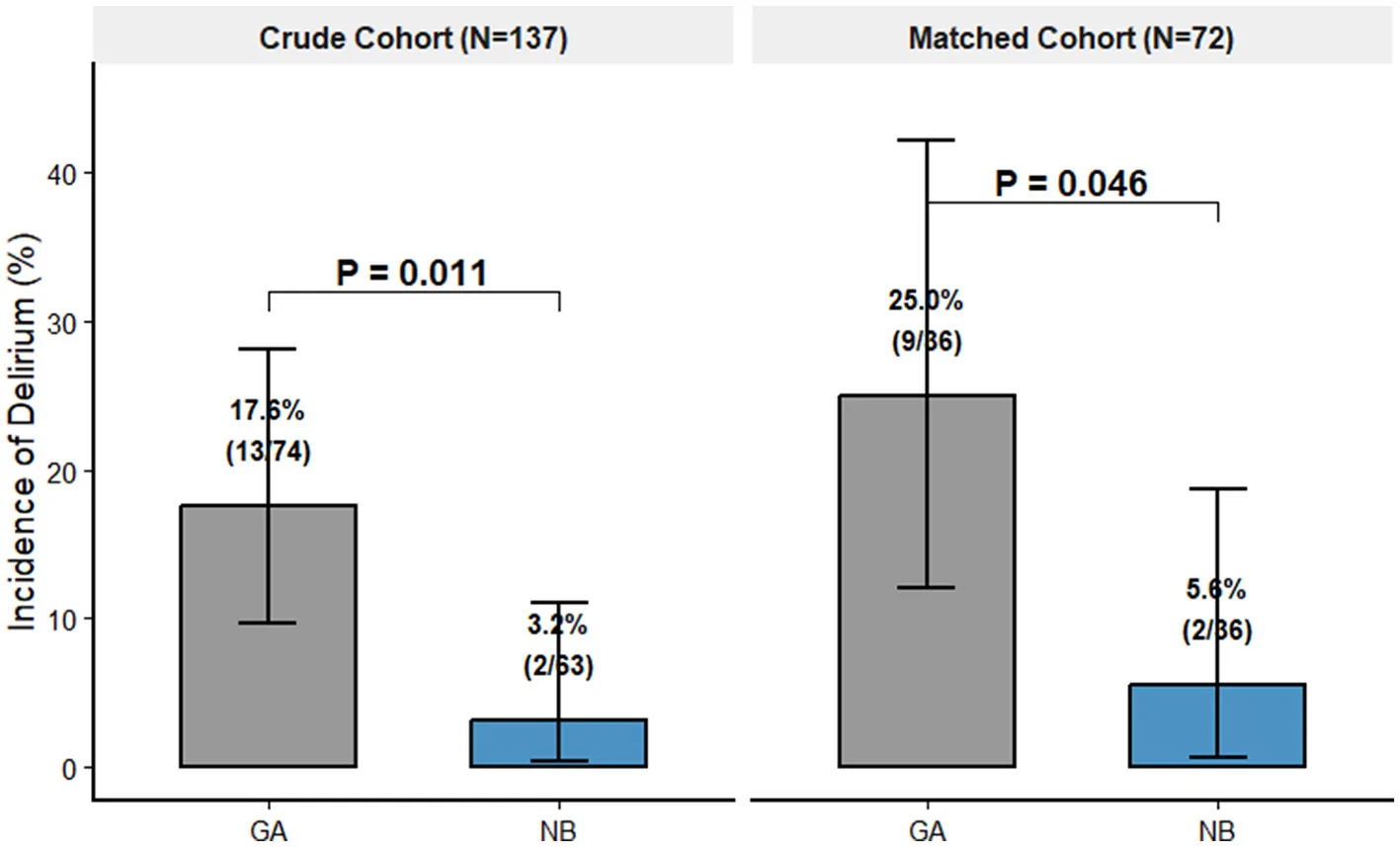
Incidence of postoperative delirium in the crude and propensity score–matched cohorts.

The incidence of postoperative delirium is illustrated in Figure 4 for both the crude and propensity score–matched cohorts. In the crude cohort, postoperative delirium occurred in 13 of 74 patients (17.6%) receiving general anesthesia, compared with 2 of 63 patients (3.2%) receiving nerve block anesthesia. This difference remained evident after propensity score matching. In the matched cohort, postoperative delirium occurred in 9 of 36 patients (25.0%) in the general anesthesia group and in 2 of 36 patients (5.6%) in the nerve block group. As illustrated in Figure 4, both the direction and magnitude of the protective association with nerve block (NB) were consistent across the crude and propensity score-matched cohorts. In the matched cohort specifically, the incidence of POD was significantly lower in the NB group (5.6%) compared to the GA group (25.0%, *P* = 0.046).

Delirium events predominantly manifested within the first three postoperative days and were distributed across a broad spectrum of ages and metabolic control levels (HbA1c), reinforcing the multifactorial etiology of POD in the diabetic foot population.

### Association between anesthesia technique and delirium risk

In analyses examining the association between anesthesia technique and postoperative delirium across different analytical models (Figure 1), nerve block anesthesia was associated with a markedly lower odds of postoperative delirium compared with general anesthesia in the crude cohort. This association remained statistically significant after multivariable adjustment for age, glycated hemoglobin, ASA physical status, and operative duration in the full cohort. In the propensity score–matched cohort, nerve block anesthesia continued to demonstrate a protective association with delirium, with effect estimates closely aligned with those obtained from the adjusted analysis of the crude cohort. As shown in Figure 1, odds ratios were consistent in direction and magnitude across the crude, multivariable-adjusted, and propensity score-matched models, confidence intervals overlapped substantially, and none of the models suggested an increased risk of delirium associated with nerve block anesthesia. This concordance across analytical strategies supports the robustness of the association notwithstanding the residual covariate imbalance noted above.

### Exploratory evaluation of perioperative factors

To explore potential factors contributing to the observed association, perioperative variables were examined in relation to delirium occurrence. Patients who developed delirium required higher intraoperative opioid doses than those who did not, regardless of anesthesia technique. In contrast, postoperative inflammatory markers exhibited substantial interindividual variability and were not consistently associated with delirium status. These findings suggest that reduced systemic opioid exposure may represent one plausible pathway through which nerve block anesthesia is associated with a lower delirium risk, whereas early postoperative inflammatory markers may be less informative within routine clinical sampling intervals.

### Summary of principal findings

Taken together, and as illustrated in Figures 1–4, ultrasound-guided peripheral nerve block anesthesia was associated with a significantly lower incidence of postoperative delirium compared with general anesthesia in patients undergoing surgery for diabetic foot disease. This association remained directionally stable across crude, multivariable-adjusted, and propensity score-matched analyses and was accompanied by a meaningful reduction in opioid exposure without evidence of increased operative complexity or adverse perioperative effects.

## Discussion

Postoperative delirium (POD) represents the acute component of perioperative neurocognitive disorders (PND) and is increasingly framed within a standardized nomenclature to improve comparability across studies and clinical pathways (Evered et al., 2018). Consistent with contemporary perioperative practice reviews and consensus guidance, POD is understood as a multifactorial syndrome driven by the interaction of baseline vulnerability and perioperative precipitating insults, with limited definitive pharmacologic treatments once established—thereby shifting clinical emphasis toward risk stratification and prevention bundles (Feng et al., 2024; Jin et al., 2020; Veronese et al., 2024; Peden et al., 2021). In the current cohort, ultrasound-guided peripheral nerve block anesthesia was associated with a lower incidence of POD than general anesthesia after robust confounding control, and the directionality of effect remained consistent across analytical strategies, supporting internal coherence of the findings.

A biologically plausible explanation centers on neuroinflammation and immune-to-brain signaling. Surgery and anesthesia can activate peripheral inflammatory cascades, disrupt blood–brain barrier integrity, and amplify microglial activation, ultimately perturbing attention and arousal networks that characterize delirium phenotypes (Subramaniyan and Terrando, 2019; Xiao et al., 2023). Recent synthesis work emphasizes that “surgery-induced inflammation” is a convergent pathway in POD pathogenesis and provides the mechanistic basis for multicomponent preventive approaches that attenuate surgical stress, optimize physiologic homeostasis, and minimize deliriogenic exposures (Feng et al., 2024; Subramaniyan and Terrando, 2019; Xiao et al., 2023). Within this framework, peripheral nerve blocks may reduce nociceptive input and stress response while limiting systemic sedative-hypnotic exposure, which could translate into a lower delirium propensity in vulnerable patients. The dissociation between suppressed IL-6 levels and unchanged CRP concentrations in the NB group highlights the distinct temporal kinetics of these biomarkers. IL-6 serves as a proximal “trigger” in the immune-to-brain signaling axis, reflecting acute surgical stress more sensitively than CRP, which is a downstream, delayed hepatic response. This suggests that NB's primary mechanistic advantage lies in attenuating the early inflammatory cascade that directly precedes microglial activation (Jin et al., 2020; Xiao et al., 2023; Lan et al., 2024).

The absence of a significant between-group difference in postoperative CRP levels is best understood within the temporal biology of the acute-phase response. CRP is synthesized by the liver in response to upstream IL-6 signaling, with circulating levels typically peaking 48–72 h after a surgical insult—a time course that substantially lags behind the early cytokine surge captured at our 24-h measurement point. Consequently, a single CRP measurement at 24 h postoperatively may be insufficient to detect differential attenuation of the hepatic acute-phase response between anesthesia groups, even when upstream IL-6 modulation is already evident. Future studies incorporating serial CRP measurements across postoperative days 1–3 would offer greater resolution to determine whether the early IL-6 suppression observed with NB ultimately translates into a reduced CRP trajectory. Similarly, the lack of a significant difference in length of hospital stay likely reflects the multifactorial and non-clinical determinants of discharge timing in a diabetic foot population, where factors such as wound care requirements, the availability of outpatient nursing support, institutional discharge planning protocols, and family or social support capacity often dominate over anesthetic technique in determining the duration of inpatient stay. Given that POD itself was a relatively infrequent event in our matched cohort, the statistical power to detect between-group differences in downstream outcomes such as length of stay was limited; adequately powered prospective studies with pre-specified secondary endpoints would be required to assess this relationship rigorously.

Analgesia-related pathways are also highly relevant. Contemporary perioperative reviews highlight a consistent association between higher postoperative pain burden and delirium risk, while emphasizing the clinical trade-off that both inadequate analgesia and excessive opioid exposure can be deliriogenic (Jin et al., 2020; Khaled et al., 2025). This aligns with broader evidence showing dose-dependent delirium risk associated with opioids in critically ill populations and supports an “opioid-sparing” target as a practical preventive lever (Duprey et al., 2021). The opioid-sparing effect of NB may provide a dual benefit: it minimizes direct deliriogenic drug exposure and may help preserve postoperative sleep architecture. Since high-dose opioids are known to induce REM sleep deprivation and sleep fragmentation—both potent drivers of delirium—the superior regional analgesia provided by NB may promote a more resilient circadian rhythm in the early recovery phase (Jin et al., 2020; Khaled et al., 2025; Duprey et al., 2021; Lan et al., 2024). Importantly, this analgesia-centered interpretation does not require claiming a uniform reduction in systemic inflammation; rather, it is compatible with a model in which pain control quality, sedation depth, and opioid dosing interact to shape delirium risk in the early postoperative period (Jin et al., 2020; Khaled et al., 2025; Lan et al., 2024).

The observed benefit of NB may be amplified in the diabetic foot population due to the pre-existing neurovascular unit impairment and blood–brain barrier (BBB) vulnerability inherent in chronic hyperglycemia. In these patients, the anesthetic technique may not merely provide analgesia but could serve as a “neuro-protective” strategy by shielding a sensitized central nervous system from the secondary hit of systemic inflammatory surges. Meta-analytic evidence indicates that diabetes is associated with higher odds of delirium, albeit with heterogeneity, suggesting that metabolic milieu, vascular disease burden, and medication profiles may interact with perioperative triggers (Komici et al., 2025; Itami et al., 2024). Evidence specific to diabetic foot procedures is emerging: across distal extremity procedures, regional and local anesthetic techniques have been reported as feasible and effective alternatives to general anesthesia, with signals toward improved perioperative profiles in patients undergoing diabetic foot ulcer surgery and below-knee operations (Zhu et al., 2023; Zou et al., 2024; Rich et al., 2024; Kocayigit et al., 2023). In such settings, the anesthetic choice may function as a modifiable precipitating factor layered onto baseline metabolic and vascular vulnerability, reinforcing the rationale for individualized anesthetic planning as part of delirium prevention.

At the same time, the broader literature on “regional vs. general” anesthesia and delirium is not uniform, and this nuance should be explicitly acknowledged. Randomized evidence in hip fracture surgery has not demonstrated a significant reduction in POD with neuraxial vs. general anesthesia: the multicenter RAGA trial reported no significant difference in delirium between regional anesthesia (without sedation) and general anesthesia (Li et al., 2022), and the multicenter REGAIN trial similarly found no significant difference in delirium or functional recovery between spinal and general anesthesia for hip surgery in older adults (Neuman et al., 2021). Several population-level and procedural differences may help account for the apparent discrepancy with our findings. First, hip fracture surgery in these trials was predominantly performed under spinal (neuraxial) anesthesia, which produces dense sensory and motor blockade through a central mechanism and carries distinct hemodynamic effects—including sympathetic blockade and systemic hypotension—that differ fundamentally from the targeted peripheral nerve blockade used in our study. Peripheral nerve blocks in the lower extremity spare central autonomic regulation, reduce the likelihood of intraoperative hypotension, and avoid the sedative co-medications often required for patient comfort during neuraxial procedures. Second, hip fracture patients frequently present with higher frailty scores, acute physiological derangement secondary to the fracture itself, and greater surgical complexity and blood loss than patients undergoing elective or semi-elective diabetic foot procedures; the magnitude of the surgical stress insult and its inflammatory sequelae therefore differ substantially between these populations. Third, in diabetic foot patients, pre-existing peripheral neuropathy and vascular compromise may amplify the systemic neuroinflammatory response to surgical stimuli, creating a setting in which the local anti-nociceptive and opioid-sparing properties of peripheral nerve block are more impactful relative to the patient's baseline vulnerability than they would be in a less metabolically compromised population. Taken together, these considerations suggest that it would be inappropriate to extrapolate null findings from hip fracture RCTs to diabetic foot surgery, and support the design of population-specific trials to resolve this question. Meta-analyses and systematic reviews similarly report heterogeneous results depending on surgical population, delirium assessment methods, sedation practices, and baseline risk distribution (Fanelli et al., 2022; Lin et al., 2023; Guo et al., 2023; Kim et al., 2023).

These findings suggest that delirium risk is not determined solely by the categorical label of anesthesia type, but by the composite perioperative exposure profile—sedation depth, hemodynamic stability, analgesia quality, anticholinergic burden, sleep disruption, early mobilization, and delirium screening frequency—elements that are strongly emphasized in contemporary guidelines and prevention strategies (Feng et al., 2024; Jin et al., 2020; Veronese et al., 2024; Lan et al., 2024; Peden et al., 2021). Within this interpretation, the observed association in diabetic foot surgery may reflect an anesthesia strategy that effectively concentrates several protective exposures (opioid-sparing analgesia, reduced hypnotic requirement, stable physiology) rather than implying that regional techniques universally prevent delirium across all surgeries.

Consistency with guideline recommendations further supports clinical relevance. The 2024 ESAIC guideline update underscores the preventable nature of POD, recommends validated screening, and promotes multicomponent perioperative prevention—including optimizing analgesia, minimizing deliriogenic drugs, and implementing structured postoperative care (Feng et al., 2024). Similarly, perioperative brain health initiatives consolidate practical tools for risk identification and preventive workflows, strengthening the rationale for anesthesia strategies that facilitate opioid-sparing and multimodal perioperative management (Peden et al., 2021). In this context, peripheral nerve block anesthesia can be positioned not as a standalone “delirium intervention,” but as a key component within a broader prevention bundle that aligns with guideline-driven best practices (Feng et al., 2024; Jin et al., 2020; Lan et al., 2024; Peden et al., 2021). Emerging non-pharmacological interventions targeting cortical excitability—such as preoperative repetitive transcranial magnetic stimulation over the left dorsolateral prefrontal cortex—have also demonstrated significant reductions in POD incidence in elderly patients undergoing elective non-cardiac surgery (Gao et al., 2026), further underscoring that anesthetic optimization and neuromodulatory strategies may serve as complementary components within a comprehensive perioperative brain protection framework.

Several limitations warrant careful interpretation. First, the retrospective design cannot entirely preclude confounding by indication, where unmeasured factors such as preoperative cognitive reserve or intraoperative blood pressure volatility might have influenced anesthetic choice and POD risk. Consistent with this, propensity score matching achieved balance on categorical covariates but left a residual imbalance in preoperative CRP (SMD = 0.83) and smaller residual differences in age, body mass index, and operative duration. Notably, preoperative CRP was higher in the general anesthesia group, so this residual imbalance could in principle bias the comparison toward a higher delirium rate in that group; we therefore cannot exclude that it contributed to the observed association. The protective association was nonetheless directionally and quantitatively consistent across the crude, multivariable-adjusted, and matched models (Figure 1), but the matched estimates should be interpreted alongside these complementary analyses rather than in isolation, and residual confounding cannot be fully excluded. Second, delirium events were relatively infrequent, limiting precision for subgroup exploration and raising the possibility that unmeasured factors (e.g., baseline cognitive status not captured, intraoperative depth of anesthesia monitoring, postoperative sedative exposure, sleep disruption, or differential mobilization practices) influenced outcomes (Feng et al., 2024; Jin et al., 2020; Veronese et al., 2024; Lan et al., 2024). Third, delirium assessors were not blinded to the anesthesia technique received by each patient, which may have introduced ascertainment bias, particularly for ambiguous presentations at the threshold of formal CAM criteria; because of the retrospective design, blinded prospective assessment was not feasible. Fourth, delirium was assessed once daily in the morning, which may have led to underestimation of the true incidence of postoperative delirium, as transient or nocturnal episodes—particularly of the hypoactive subtype, which represents the most common and frequently under-recognized form—can be missed by single daily evaluations; because this ascertainment limitation applies equally to both groups, any resulting underestimation is expected to be non-differential and would likely attenuate rather than exaggerate the observed protective effect. Fifth, the PSM process resulted in retention of 72 of the original 137 patients (52.6%), primarily attributable to the unequal original group sizes (GA: *n* = 74, NB: *n* = 63) and residual heterogeneity in preoperative CRP that constrained available matches within the predefined caliper; the directional and magnitude consistency of effect estimates across the matched and unmatched adjusted analyses (Figure 1) nonetheless supports the internal robustness of the principal findings. Sixth, cognitive status was not prospectively assessed using validated instruments such as the MMSE or MoCA; the exclusion of severe cognitive impairment was based on existing clinical documentation, and patients with mild or undiagnosed preoperative cognitive impairment cannot therefore be entirely excluded as a source of residual confounding. Finally, reliance on routine inflammatory markers may be insufficient to map the neuroinflammatory mechanisms of delirium; more granular biomarker profiling and standardized delirium screening frequency could strengthen mechanistic inference in future studies (Feng et al., 2024; Subramaniyan and Terrando, 2019; Xiao et al., 2023; Lan et al., 2024).

Future work should prioritize prospective, adequately powered, multicenter trials in diabetic foot surgery to determine whether peripheral nerve block–based strategies reduce POD under standardized delirium assessment and modern perioperative pathways. Such studies should embed process measures (opioid dosing trajectories, sedation depth, sleep metrics, early mobilization, and delirium prevention bundle adherence) to clarify which exposure components drive any observed benefit and to maximize implementation value (Feng et al., 2024; Jin et al., 2020; Khaled et al., 2025; Veronese et al., 2024; Lan et al., 2024; Peden et al., 2021).

## Conclusion

Ultrasound-guided peripheral nerve block anesthesia was associated with a lower incidence of postoperative delirium than general anesthesia in patients undergoing diabetic foot surgery, and this association remained directionally consistent across multivariable adjustment for key perioperative confounders and propensity score matching. The finding is biologically plausible within current models of delirium pathogenesis, consistent with potential mediation through opioid-sparing analgesia and reduced exposure to potentially deliriogenic perioperative factors, and aligns with guideline-supported multicomponent prevention principles. However, given the retrospective design, limited event counts, residual covariate imbalance, and the known heterogeneity of evidence across surgical populations, prospective multicenter studies with standardized delirium assessment and detailed perioperative process measures are required to confirm causality and define the most effective, scalable prevention strategy.

## Data Availability

The raw data supporting the conclusions of this article will be made available by the authors, without undue reservation.
